# Multimodal parameter spaces of a complex multi-channel neuron model

**DOI:** 10.3389/fnsys.2022.999531

**Published:** 2022-10-20

**Authors:** Y. Curtis Wang, Johann Rudi, James Velasco, Nirvik Sinha, Gideon Idumah, Randall K. Powers, Charles J. Heckman, Matthieu K. Chardon

**Affiliations:** ^1^Department of Electrical and Computer Engineering, California State University, Los Angeles, Los Angeles, CA, United States; ^2^Department of Mathematics, Virginia Tech, Blacksburg, VA, United States; ^3^Interdepartmental Neuroscience, Northwestern University, Chicago, IL, United States; ^4^Department of Mathematics, Case Western Reserve University, Cleveland, OH, United States; ^5^Department of Physiology and Biophysics, University of Washington, Seattle, WA, United States; ^6^Department of Neuroscience, Northwestern University, Chicago, IL, United States; ^7^Physical Medicine and Rehabilitation, Shirley Ryan Ability Lab, Chicago, IL, United States; ^8^Physical Therapy and Human Movement Sciences, Northwestern University, Chicago, IL, United States; ^9^Northwestern-Argonne Institute of Science and Engineering, Evanston, IL, United States

**Keywords:** computational neuroscience, parameter estimation, model fitting, Hodgkin–Huxley, Bayesian framework, Markov chain Monte Carlo, multimodal posterior

## Abstract

One of the most common types of models that helps us to understand neuron behavior is based on the Hodgkin–Huxley ion channel formulation (HH model). A major challenge with inferring parameters in HH models is non-uniqueness: many different sets of ion channel parameter values produce similar outputs for the same input stimulus. Such phenomena result in an objective function that exhibits multiple modes (i.e., multiple local minima). This non-uniqueness of local optimality poses challenges for parameter estimation with many algorithmic optimization techniques. HH models additionally have severe non-linearities resulting in further challenges for inferring parameters in an algorithmic fashion. To address these challenges with a tractable method in high-dimensional parameter spaces, we propose using a particular Markov chain Monte Carlo (MCMC) algorithm, which has the advantage of inferring parameters in a Bayesian framework. The Bayesian approach is designed to be suitable for multimodal solutions to inverse problems. We introduce and demonstrate the method using a three-channel HH model. We then focus on the inference of nine parameters in an eight-channel HH model, which we analyze in detail. We explore how the MCMC algorithm can uncover complex relationships between inferred parameters using five injected current levels. The MCMC method provides as a result a nine-dimensional posterior distribution, which we analyze visually with solution maps or landscapes of the possible parameter sets. The visualized solution maps show new complex structures of the multimodal posteriors, and they allow for selection of locally and globally optimal value sets, and they visually expose parameter sensitivities and regions of higher model robustness. We envision these solution maps as enabling experimentalists to improve the design of future experiments, increase scientific productivity and improve on model structure and ideation when the MCMC algorithm is applied to experimental data.

## 1. Introduction

Systematic parameter exploration is an unmet need for model building in neuroscience. Complete specification of models in neuroscience or systems physiology requires identification of several parameters. For single neurons, these typically include geometric and electrical properties of the cell body, dendrites, and axons. Even with the sophistication of modern experimental techniques, however, measuring all the necessary parameters is almost always impossible. Additionally, many single-neuron properties exhibit remarkable context-dependent variability, even within the same animal. For example, changes in the neuromodulatory environment alter the spiking dynamics of spinal motoneurons (Heckman et al., 2009) and thalamocortical neurons (Pape and McCormick, 1989) by influencing their ionic conductances. Thus, any single measurement at one time instance would be insufficient to inform all single-neuron/network properties because it cannot account for the wide spectrum of behaviors observed *in vivo*.

To complicate matters further, redundancy in biological systems leads to similar activity profiles that can be produced by many different neurons or neuronal networks with dissimilar properties (Swensen and Bean, 2005; Schulz et al., 2007; Roffman et al., 2012). For example, lateral pyloric neurons in the crab stomatogastric ganglion exhibit as much as two- to four-fold interanimal variability in three different ion channel densities and their corresponding mRNA levels (Schulz et al., 2006). Therefore, even averaging a parameter from multiple experimental preparations may fail to generate the desired behavior in the computational models constructed from them (Golowasch et al., 2002). Systematic exploration of the parameter space is obligatory to fit a neuron model to experimental data. Additionally, this systematic exploration may reveal deeper insights and motivate future experiments by unraveling undiscovered parameter combinations that might reproduce the same experimentally observed behavior.

Fitting experimental data to neuron models has been a major challenge for neuroscientists. In fact, the parameters of the first biologically realistic quantitative description of the neuronal membrane were hand fitted by Hodgkin and Huxley (1952). This approach has remained popular; for example, the peak conductances of ion channels in a model of an elemental leech heartbeat oscillator were hand tuned by Nadim et al. (1995). Likewise, both the single compartment and network parameters of a small group of neurons used to model the crustacean pyloric network were hand tuned by Soto-Trevino et al. (2005) to reproduce a variety of experimentally observed behaviors. However, the ever-increasing dimensionality and complexity of neuron model (Hodgkin and Huxley's description contained only three membrane currents), accompanied by the concomitant increase in computational resources, have made hand tuning of parameters a near infeasible task. Besides, hand tuning of parameters also introduces potentially undesired experimenter bias, because different parameters are assigned preconceived roles during the tuning process (Van Geit et al., 2008). Nevertheless, manual exploration of parameter sets may be unavoidable because the initial range of values of different parameters over which the automated search is to be performed must be determined by the experimenter based on physiological constraints.

### 1.1. Computational inference for neural models

The body of research on parameter estimation for models of neural dynamics for single cells or circuits spans across various scientific communities, approaches, and neuron models. A gap exists, however, between researchers with rich physiological knowledge, on the one hand, and researchers working on new solution algorithms for inference, on the other hand. This gap presents opportunities to create a bridge between physiologists, engineers, computational scientists, and mathematicians by exploring existing and developing new inference techniques for neuron dynamics of single cells and circuits.

#### 1.1.1. Multiple realizability: The dilemma of many truths

Single-neuron activities that are amenable to easy measurement (e.g., spike trains, voltage traces, local field potentials) can often be identical for different parameter combinations. Just as this situation renders experimental determination of parameter values infeasible, it also poses an incredible challenge to systematically infer a model from experimental data. For example, Prinz et al. (2004) found indistinguishable network activity from widely disparate deterministic models. Similarly, Amarasingham et al. (2015) reported statistically indistinguishable spike train outputs for different statistical processes that model the firing rate of networks. Hartoyo et al. (2019) stressed that, for models of dynamical systems, very different parameter combinations can generate similar predictions/outputs. Additionally, the authors showed that sensitivities of predicted parameters can exhibit wide variability, leading to the conclusion that modeling in neuroscience is confronted by the major challenge of identifiability of model parameters. The demonstrative model used in our present work shows a bursting neuron producing similar neural activities from multiple sets of conductance densities (Alonso and Marder, 2019).

#### 1.1.2. Approaches for estimating parameters

Computational approaches for estimating parameters of neuron models based on ordinary differential equations (ODEs) include (brute-force) grid search (Prinz et al., 2003) as well as more advanced techniques using heuristics or trial-and-error approaches, which may consist of intricate sequences of regression steps (Achard and De Schutter, 2006; Van Geit et al., 2007, 2008; Buhry et al., 2011). The latter include simulated annealing, differential evolution, and genetic algorithms. These approaches can have the disadvantage of slowly converging to an optimal set of parameters and thus becoming computationally expensive. Using gradient-based optimization to accelerate convergence (Doi et al., 2002; Toth et al., 2011; Meliza et al., 2014), on the other hand, can suffer from the non-convexity and the strong non-linearities in the objective manifold and require good initial guesses in order not to stagnate in local minima (thereby resulting in suboptimal parameter sets). Another shortcoming of recovering only a single set of parameters (also called point estimates) is the lack of knowledge about the uncertainties or error bounds for the inferred parameters. These can be obtained with a subsequent sensitivity analysis and, as a consequence, require employing additional algorithms and computational resources.

Recently the use of machine learning techniques based on artificial neural networks (ANNs) became popular. For instance, in the context of the FitzHugh–Nagumo model (Rudi et al., 2021), an ANN was constructed and optimized to generate an inverse map that is able to predict model parameters from observational data. Bittner et al. (2021) developed generative models from deep learning. Retrieving associated uncertainties for inferred parameters has been performed by Gonçalves et al. (2020), using normalizing flows with Gaussian mixtures in order to generate approximations of posterior distributions for Hodgkin–Huxley-based inverse problems. However, machine learning techniques require generating large sets of data for training the artificial neural networks, where each training sample entails the numerical solution of the ODE system of the neuron model.

Theoretical neuroscientists have been developing and utilizing statistical methods for inference and uncertainty quantification (Van Geit et al., 2007; Vavoulis et al., 2012) and have been relying on Bayesian inference frameworks (Ahmadian et al., 2011; Doruk and Abosharb, 2019) in order to estimate parameters and quantify uncertainties of the recovered parameters. The uncertainties in recovered parameters with Bayesian inference frameworks are represented by posterior density functions, which describe a “landscape” of more likely parameters (“peaks in the landscape”) and less likely parameters (“valleys in the landscape”; see Section methods for details). Bayesian likelihoods or their approximations are constructed, which in turn enables direct maps to posterior densities without numerical solutions of neuron models to evaluate objective (or loss) functions. Chen (2013) provides an overview of Bayesian methods for neural spike train analysis. René et al. (2020) and Schmutz et al. (2020) have utilized Markov chain Monte Carlo for inference from spike train data of population models.

### 1.2. Using Markov chain Monte Carlo (MCMC) algorithms for inference

MCMC algorithms play a critical role in our approach for parameter estimation. For a brief introduction and background information about MCMC algorithms, we refer to the Section methods.

#### 1.2.1. Benefits and limitations of using MCMC for parameter searches

One of the main benefits of using MCMC is the detailed picture or “landscape” of the posterior density that it can provide. By analyzing the posterior, the uncertainty in the inferred parameters can be quantified, and the dependencies between different parameters in the model can be identified. Another benefit is that MCMC does not require derivatives of neuron models or the loss function (measuring misfit of the data and model output). This is an important property in the case of the complex model and non-differentiable loss function used by Alonso and Marder (2019), which our present work utilizes. We propose to utilize a specific MCMC method called parallel tempering MCMC. It has the key advantage of efficiently exposing multimodality in the posterior (see Section methods for more details), which other MCMC methods can potentially leave undiscovered.

As with many other computational methods for parameter estimation, the efficiency scaling with the number of parameters (dimensionality) is a potential limitation with MCMC-based methods. Generally speaking, as the number of parameters increase, the dimensionality of the search space increases, and more iterations of MCMC may be required, leading to a longer total computation time. In the inference that we are targeting in this work, the number of inferred parameters remains at amounts where these limitations do not occur for parallel tempering MCMC. We note here that parallel tempering MCMC is able to find the multimodal posterior successfully where many other Bayesian inference methods, including Approximate Bayesian Computing with Sequential Monte Carlo, could not. Future work will determine the dimensionality limits of this computational method.

#### 1.2.2. Applications of MCMC in the context of biological modeling

In fields related to neuroscience, MCMC-based methods have been a popular choice for solving inverse problems in a Bayesian framework, where one is interested in uncertainties in addition to optimal solutions (Smith, 2013). In the context of dynamic systems in biology, MCMC techniques have been successfully employed, as described in review articles by Ballnus et al. (2017) and Valderrama-Bahamo (2019). Moreover, tempering MCMC methods have been shown to recover the multimodality of solutions in systems biology (Caranica et al., 2018; Gupta et al., 2020). However, in the context of neural dynamics with complex Hodgkin–Huxley models, such as in Alonso and Marder (2019), on which we built this work, tempering MCMC methods have not been attempted to our knowledge.

### 1.3. Contributions of this work

In this study, we address the issue of multiple realizability of specific models in neuroscience. In particular, we present the computational inference for one three-channel and one eight-channel HH neuron model with a parallel tempering MCMC algorithm as the method for inference. We successfully estimate the parameters in these models, describe the multimodality in the parameter space, and quantify the parameters' uncertainties. Additionally, we present visualizations of the high-dimensional inference solutions taking the form of posterior densities.

We discuss how these multimodal posteriors (solution maps) are produced by this method for large parameter dimensions. While the posteriors are inherently tied to both the chosen neuron model and chosen loss function, we believe, this method could allow an experimentalist the ability to explore different desired features and models.

As an alternative to manually adjusting the metric measuring fitness between data and model outputs, we present inference setups that use different stimulus currents to further constrain the algorithmically recovered parameters obtained with MCMC.

## 2. Methods

We design the parameter estimation problem in a Bayesian framework: (i) parameter values of the neuron model are treated as (multivariate) probability distributions rather than the solution being a single optimal parameter set; and (ii) the posterior distributions of parameter values can exhibit dependencies between each other, rather than assuming each parameter to be independent from others. Note that the Bayesian framework does not assume that a single independent optimum can be reached. The density of the posterior distribution is proportional to the product of a *likelihood* and a *prior* term. The likelihood is given implicitly in the form of a loss function between observational data and model output, hence requiring the numerical solution of the model for each new set of parameters. Whereas the prior is a known density function (e.g., a Gaussian with known mean and covariance) and is provided from knowledge about the parameters of the model. As a solution algorithm we utilize an extension of MCMC, the parallel tempering MCMC method, which is critical for recovering distributions of parameter sets that are Subsequently, we introduce the HH-based neuron models that we consider for computational inference.

### 2.1. Inference of parameters in a three-channel Hodgkin-Huxley neuron model

We introduce the algorithmic approach of our choice for solving an inverse problem involving a neural model. We utilize the parallel tempering MCMC method, which is described in detail below. To illustrate the type of solutions that parallel tempering MCMC can deliver, we first consider a three-channel (Na, K, and leak channels) Hodgkin-Huxley model, which is a classic and well-known conductance-based model (Hodgkin and Huxley, 1952; Mainen et al., 1995). While this model is less complex than the eight-channel model we focus on subsequently, this simpler model nevertheless exhibits the key difficulties and challenges of parameter estimation in neural dynamics. The mean squared error (MSE) between voltage traces from the model and simulated observational data serves as the loss function to measure the fit between model outputs and data for this example. To set up the inverse problem, we simulate the voltage trace for a given set of sodium and potassium conductances, denoted as the “true” parameter set, which is *g*_*Na*_ = 200 *pS*/μ*m*2 for sodium and *g*_*K*_ = 50 *pS*/μ*m*2 for potassium. This simulated voltage trace generated by the HH model serves as the data for our inverse problem; and the sodium and potassium conductances are the parameters that we aim to infer. The numerical results of the inference are presented in Section results.

### 2.2. Inference of parameters in an eight-channel Hodgkin–Huxley neuron model

The model that this work focuses on is the bursting neuron model from Alonso and Marder (2019). The model is a system of ODEs describing the time evolution of the membrane potential voltage coupled with kinetic equations for the eight voltage-gated conductances; for more details, see Equations (5–7) of Alonso and Marder (2019). We choose to analyze specifically this model because of its recently documented challenges with regards to parameter estimation, multiple candidate solutions, and complex dependencies of parameters on one another. We select the parameter values for the ground truth to be from “Model A” of Alonso and Marder (2019) for consistency. The definitions, units, and values used to generate ground truth voltage traces of the nine parameters that we seek to infer are in Table 1.

**Table 1:** Definition, values, and unit of the parameters that we seek to infer. Details for these definitions can be found in Alonso and Marder (2019) and Liu et al. (1998).

**Parameter**	**Definition**	**Ground truth value**	**Unit**
*g*_*Na*_	Max Conductance for: Fast transient *Na*^+^ current	1076.392	μ*S*
*g*_*CaT*_	Max Conductance for: Fast transient *Ca*^2+^ current	6.4056	μ*S*
*g*_*CaS*_	Max Conductance for: Slow *Ca*^2+^ current	10.048	μ*S*
*g*_*A*_	Max Conductance for: Fast transient *K*^+^ current	8.0384	μ*S*
*g*_*KCa*_	Max Conductance for: *Ca*^2+^ dependent *K*^+^ current	17.584	μ*S*
*g*_*Kd*_	Max Conductance for: Delayed rectifier *K*^+^ current	124.0928	μ*S*
*g*_*H*_	Max Conductance for: Hyperpolarization-activated inward cation current	0.11304	μ*S*
*g*_*L*_	Max Conductance for: Leak current	0.17584	μ*S*
τ_*ca*_	Time constant of *Ca*^2+^ concentration process	653.5	*ms*

The voltage traces of the AM model are obtained numerically with the BDF and LSODA solvers for ODEs from the SciPy library (Petzold, 1983; Virtanen et al., 2020). BDF and LSODA were used because of their overall convergence performance compared with other ODE solvers for stiff biological ODE models; for instance, LSODA has been analyzed for biological systems in Städter et al. (2021). The voltage trace generated by the ground truth parameters is shown in Figure 7 in orange color. The output models of this work are available in ModelDB with accession number 267583.

### 2.3. Loss function for measuring fit between data and model outputs

The main loss function used in the present study involving the AM model is the loss of Equation (4) of Alonso and Marder (2019), namely,


$$\begin{array}{l} E\left(g\right)=\alpha E_{f}+\beta E_{dc}+\gamma E_{mid}+\delta E_{sw}+\eta E_{lag}, \end{array}$$

where we use the following coefficients: α = 1, 000, β = 1, 000, and γ = δ = η = 0. *E*_*f*_ is the mismatch of the inter-burst frequency (frequency between bursts), *E*_*dc*_ is the mismatch of the inter-burst duty cycle (the ratio of the time bursting vs. not bursting per cycle), *E*_*sw*_ and *E*_*mid*_ handle slow-wave mismatches, and *E*_*lag*_ is the lag between the upward crossings and downward crossings.

We purposefully eliminate several components of the loss (i.e., γ = δ = η = 0) to avoid manual penalization between certain terms in the general form of the loss function. Our study uses the same loss function with fixed coefficient values throughout all numerical experiments, because our focus is to compare inference results and it avoids possible bias. As such, our approach differs from the objectives of Alonso and Marder (2019), where the coefficients in the loss are adjusted depending on the inference setup. Here we focus on the two components of the loss function that we observed to be dominant, associated with α, the burst frequency mismatch *E*_*f*_, and with β, the duty cycle mismatch *E*_*dc*_. The intra-burst frequency mismatch and duty cycle mismatch components are visible in Figure 7. Our objective is to test the multimodality of the inverse problem with parallel tempering MCMC methods.

### 2.4. Loss function for individual and aggregate constraint

We use the loss function (detailed in Section loss function for measuring fit between data and model outputs) to solve inverse problems, where one particular injected current is present; these studies are referred to as the individual current constraint. The individual current constraint (or “individual constraint” in short) is when the loss function value for a single current injection value (i.e., one of 0.0, 0.1 nA, …) is used for measuring the fit between data and model outputs.

In addition, we carry out numerical experiments with multiple injected currents, referred to as the aggregate current constraint. The aggregate current constraint (or “aggregate constraint” in short) is when the maximal loss function value for the entire set of injected currents (i.e., {0.0, 0.1, 0.2, 0.3, 0.4} nA) is used for the misfit between data and model outputs. The aggregate current constraint is motivated by its similarity to fitting against a frequency-current (F-I) curve (Hultborn and Pierrot-Deseilligny, 1979).

### 2.5. Introduction and background to MCMC

In this work we choose the Bayesian formulation (also called Bayesian framework), which is based on Bayes' theorem:


(1) $$\begin{array}{l} P\left(\theta |y\right)=\frac{P\left(y|\theta \right)P\left(\theta \right)}{P\left(y\right)}, \end{array}$$

where θ denotes the parameters of the ODE model and *y* is the data. θ and *y* can be scalars or, more commonly, vectors. On the left-hand side of (1), *P*(θ ∣ *y*) is the unknown *posterior* (i.e., conditional probability for the model parameters θ given the data *y*). On the right-hand side of (1), *P*(*y* ∣ θ) is the known but often intractable *likelihood* (i.e., conditional probability for the data *y* given the model parameters θ). The likelihood term is responsible for measuring the fit between data and model outputs. Hence the loss function, which we detailed above, is evaluated each time the likelihood is computed. The likelihood being intractable means that it requires the solution of a model and cannot be accessed (or sampled from) directly. Further on the right-hand side, *P*(θ) is the known and typically tractable *prior* (i.e., probability of the model parameters θ). The term *P*(*y*) is called *evidence* (i.e., total probability of the data *y*); and since it is constant with respect to θ, it simply scales the posterior by a constant and can, in practice, remain unknown.

To provide an intuition, one goal of an MCMC algorithm, such as the Metropolis–Hastings algorithm (Metropolis et al., 1953; Hastings, 1970), is to find the most likely model parameters, θ, that will produce the largest posterior (the highest probability of the model parameters to reproduce the data) through sampling. Another goal is to quantify parameter uncertainties. To this end, the algorithm visits smaller locations in the posterior (i.e., lower probabilities of the model parameters to reproduce the data). However, these visits to lower-probability locations of the posterior happen adaptively and automatically at smaller frequencies. The concept of MCMC visiting locations of high probability at greater frequencies and locations of lower probability at smaller frequencies is a key property of the algorithm.

**Figure 1:**
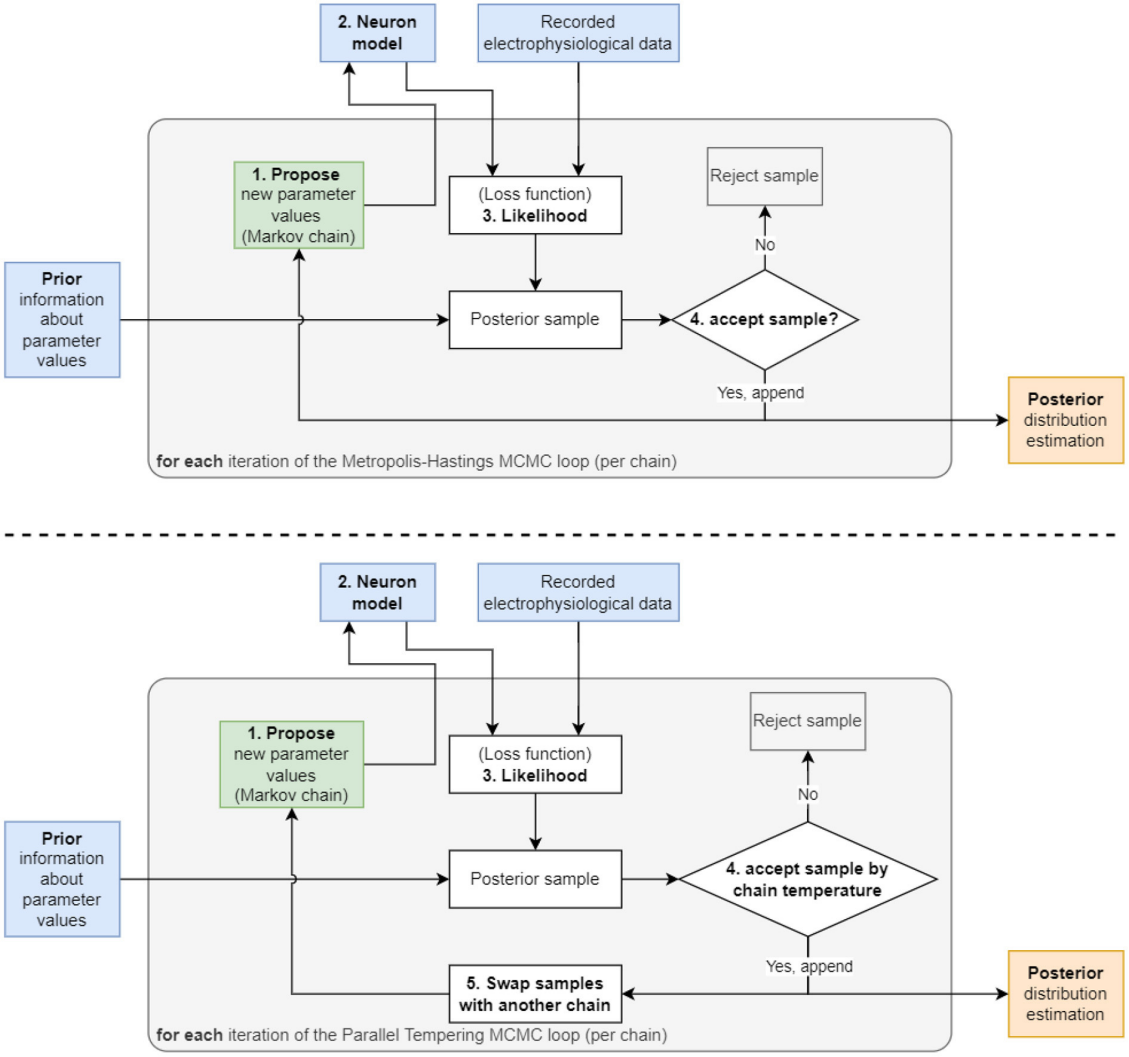
Two diagrams depicting the flow of the two algorithms used in this work. **(Top)** The Metropolis–Hastings MCMC algorithm begins with a “1. Propose” step, where the algorithm selects an initial set of parameters to be used in the “2. Neuron model” step. The model is then solved using the parameters and its output compared to recorded data to produce the “3. Likelihood”. This likelihood is then multiplied with the prior to generate the probability value used in the “4. accept sample?” step to accept or reject the sample. This cycle is repeated until the number of preset iterations is reached. **(Bottom)** The Parallel Tempering MCMC algorithm used for the 9 parameter AM model. This algorithm utilizes an additional “5. Swap samples with another chain” step to increase mixing between multiple chains and to encourage the chains to sample in more remote areas of the parameter space, hence, detect multimodality.

In detail, at each iteration of MCMC, the algorithm first proposes a new value for (one or multiple) parameters, θ_proposal_, by randomly choosing from a proposal distribution (e.g., a normal distribution centered at the value of the previous iteration θ_previous_) (Figure 1). The likelihood *P*(*y* ∣ θ_proposal_) and prior *P*(θ_proposal_) are evaluated at the proposed value and multiplied to obtain a new value of the posterior. In the second MCMC iteration, the algorithm includes the proposed θ_proposal_ in a sequence of visited points or discards it, where the aim is to more frequently keep points of higher probability. To determine whether θ_proposal_ is accepted or rejected relative to the previous θ_previous_, the algorithm takes the ratio of the proposed posterior to the current posterior. If the ratio is greater than one, θ_proposal_ must have a higher probability, and therefore this new parameter is saved because it is producing a better outcome. If the ratio is below one, then the algorithm applies a randomized rejection criterion such that some θ_proposal_ is stored at lower frequencies. Subsequently, this cycle repeats, where θ_proposal_ becomes the new θ_previous_, if acceptance was successful. Note that in this algorithm, dividing the new value by the previous value of the posterior, *P*(*y*) cancels out, thus showing that constant scaling factors are irrelevant for MCMC.

The Metropolis–Hastings algorithm constructs a sequence, also called a chain, of parameters (also called samples) that it visits during the iterations (Figure 1). This method is suitable for unimodal posterior distributions. However, it is not designed to capture multimodal posteriors (i.e., posterior with multiple peaks). Therefore, extensions of MCMC have been developed to deal with multimodal distributions. Early work by Marinari and Parisi (1992); Geyer and Thompson (1995) employed simulated tempering, which is akin to simulated annealing algorithms from the field of optimization. More recently, parallel tempering MCMC (Łacki and Miasojedow, 2016; Vousden et al., 2016) was proposed. This builds on ideas from simulated tempering and, additionally, uses multiple chains of single-chain methods, such as Metropolis–Hastings, in parallel. The key idea of tempering, which enables MCMC to discover multimodalities in posteriors, is that the posterior is taken to a power with an exponent, γ, between zero and one: 0 < γ < 1. Hence we obtain *P*^γ^(θ ∣ *y*). This has the effect that smaller locations in the posterior are elevated and can be visited more frequently by Metropolis–Hastings.

### 2.6. MCMC method for estimating parameters in AM model

This section provided details about our implementation for estimating parameters and their uncertainties in AM models. Our AM model and MCMC methods constitute the following components:

- Neuron model component: Computes voltage traces for the AM model and a given set of candidate parameters
- Likelihood component: Computes the Bayesian likelihood using the loss function based on the voltage trace obtained by neuron model component
- Prior component: Evaluates the prior based on the candidate set of parameters
- Estimator component: Proposes candidate parameters at random (the proposal sampler)

The sampler used was the adaptive parallel tempering sampler (Miasojedow et al., 2013) within the PyPESTO library (Stapor et al., 2018). Parallel tempering runs independent Markov chains at various temperatures and performs swaps between the chains. Each individual chain runs adaptive Metropolis–Hastings MCMC.

The loss functions for individual and aggregate constraints are evaluated for each candidate parameter in the MCMC algorithm within the likelihood term. The prior term is defined as a uniform distribution (i.e., unbiased prior) within predetermined boundaries, which were chosen sufficiently wide to permit physiologically relevant model parameters.

### 2.7. Numerical experiment setup

To investigate the robustness of parallel tempering MCMC on the inverse problem governed by the AM model, we use model A from Alonso and Marder (2019), which is defined by the parameters that are listed in Section inference of parameters in an eight-channel Hodgkin–Huxley neuron model, as ground truth data. We use this model to generate five voltage traces each using different injected currents: 0.0, 0.1, 0.2, 0.3, and 0.4 nA. Noise to simulate physical measurement noise was not added to the voltage trace of the ground truth, for several reasons. To have a numerical experiment setup similar to Alonso and Marder (2019), we also did not alter the ground truth trace data.

The conductances for the ion channels and the time constant for the calcium channel are the nine parameters that we target for inference. Each inference with individual current constraint consisting of running the MCMC algorithm for a predetermined number of iterations/samples. Additionally, for the inference with aggregate current constraint, a single run of MCMC was performed. Each MCMC run used 24 to 32 parallel chains and 10,000 to 14,000 samples per chain. Geweke's convergence diagnostic was used to determine the burn-in of each chain (Stapor et al., 2018). The burn-in is an initial phase of MCMC where the gathered samples do not satisfy certain statistical properties to be considered adequate samples of the posterior. In certain cases, despite parallel tempering, there are MCMC chains that can remain static through the parameter space. Results from chains that did not move sufficiently (ratio of standard deviation to mean) were filtered out in postprocessing.

### 2.8. Software improvements for accelerating parallel tempering MCMC

We reduced the computational time of the MCMC inference by carrying out various improvements of the source code. Increasing the number of Markov chains benefits the detection and exploration of multimodal posteriors; however, it also increases the computation time. To shorten the computation time, we parallelized the MCMC algorithm to run on each CPU core concurrently using multiprocessing packages (Conda-Forge Community, 2015). This approach significantly decreased computation time because of the parallel nature of the chains. The total runtime for each MCMC run was between 24 and 48 h. We utilized three different machine architectures: (1) the Broadwell nodes (36-core, 128 GB RAM) on the parallel cluster Bebop at Argonne National Laboratory, (2) a workstation equipped with a dual 16-core Intel Xeon 4216 CPU (total of 32 cores and 64 threads) with 128 GB of RAM, and (3) a workstation with a 32-core (64-thread) AMD Ryzen 3970x CPU with 128 GB of RAM.

## 3. Results

The results of our study show that our design of the inverse problem combined with parallel tempering MCMC for solving the inverse problem is able to successfully overcome the inference challenges. The algorithm can recover large numbers of parameter sets where data and model output are consistent. Parallel tempering MCMC returns posterior distributions of parameter sets, called the posterior in short, and we visualize the multidimensional posterior by showing *solution maps* in one and two dimensions where the non-visible dimensions have been integrated out (i.e., showing a marginal distribution). With these solution maps physiologists can investigate the results from MCMC in order to decide which parameters warrant more investigation from a physiological perspective. This is a main advantage of *solution maps* compared with *solution points* (i.e., a single set of “optimal” parameters) obtained from optimization algorithms. Additionally, the robustness of parameters can be assessed, because solution maps indicate by how much parameters can be perturbed while still delivering model outputs that are consistent with data.

### 3.1. Inference proof of concept with the three-channel Hodgkin–Huxley model

**Figure 2:**
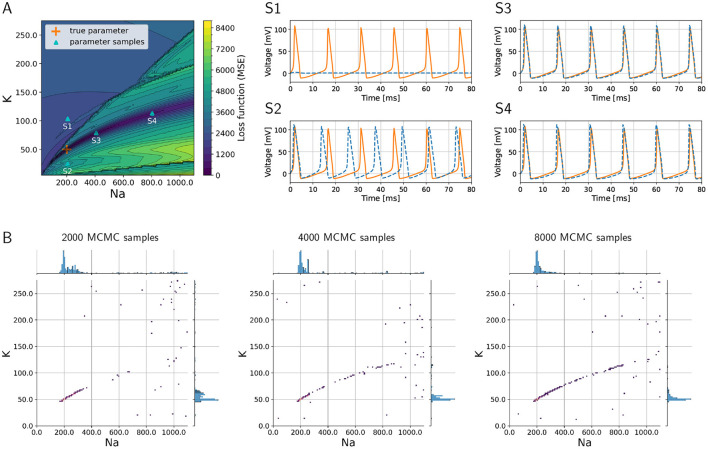
Loss function corresponding to the inverse problem with a three-channel HH model and how MCMC is able to successfully recover multiple optimal parameter sets and quantify uncertainties and parameter trade-offs. **(A)** Loss function landscape (colors) over the parameters, sodium (*g*_*Na*_) and (*g*_*K*_) potassium conductances. The “true” parameter set represents a global minimizer of the loss; the valley of the loss along points S3 and S4 (in dark blue color of loss) translates to parameter uncertainties (or trade-offs). Graphs S1–S4 depict voltage traces at corresponding points in **(A)**. **(B)** Densities of MCMC samples, where the dark purple color of highest density overlaps with the true parameter set. As the number of MCMC samples increases, the the method recovers the valley of the loss landscape A and hence quantifies uncertainties in the parameters.

As a proof of concept, we consider the inverse problem where the goal is to recover the sodium and potassium conductances in an HH model. To demonstrate the challenges of the problem, we visualize the loss function that would have to be minimized to find the optimum in Figure 2A, where the colors indicate the (positive) loss value. The true parameter set is located in a “valley” in the loss “landscape,” whereas the loss is large for parameters that produce large discrepancies between model output and data such as shown in graph S1 corresponding to point S1 in Figure 2A. However, multiple sets of parameters exist that are local minima in this landscape, and these multiple local minima pose major challenges because optimization algorithms may present one of them as the “optimal” solution. These local minima will not give a sufficiently good fit of model outputs vs. data, as is illustrated in graph S2 of Figure 2 that corresponds to point S2 in the landscape (Figure 2A). Such local minima are known to be problematic for numerical optimization algorithms (Nocedal and Wright, 2006).

Additionally, we aim to quantify the uncertainty with respect to the parameters associated with sodium and potassium conductances, and we would like to understand the sensitivities of the model with respect to these parameters. In Figure 2, the graphs S3 and S4 illustrate how different parameters produce voltage traces similar to the data, while at the same time this uncertainty in the parameters is visible in the landscape as a valley (dark blue color in Figure 2A, which corresponds to low values of the loss function).

The numerical solution of the Bayesian inverse problem with parallel tempering MCMC successfully provides a probability density spanned by the two-dimensional parameter space. The density is large, where the loss function in Figure 2A has its major valley. We show the progress of the MCMC algorithm in Figure 2B as the number of collected samples increases from 2,000 to 8,000. As the sample count (i.e., iteration count of MCMC) grows, the algorithm generates a longer tail along the valley, showing a clearer picture of the parameter uncertainties. The true parameter values are clearly visible in the high-density region as a dark blue area around 200 and 50 *pS*/μ*m*2 for sodium and potassium conductances, respectively. Furthermore, the tail to the upper right of the true parameter set shows the trade-off between sodium and potassium, when model outputs keep being consistent with data even though the values of the parameters deviate from the truth.

These results serve as a proof of concept that the parallel tempering MCMC algorithm can successfully tackle multimodal losses. Next we transition to a more complex model for neural dynamics, which is the focus and the main result of the present work.

### 3.2. Inference with the complex eight-channel Alonso–Marder (AM) model

#### 3.2.1. AM model—Posterior distribution

**Figure 3:**
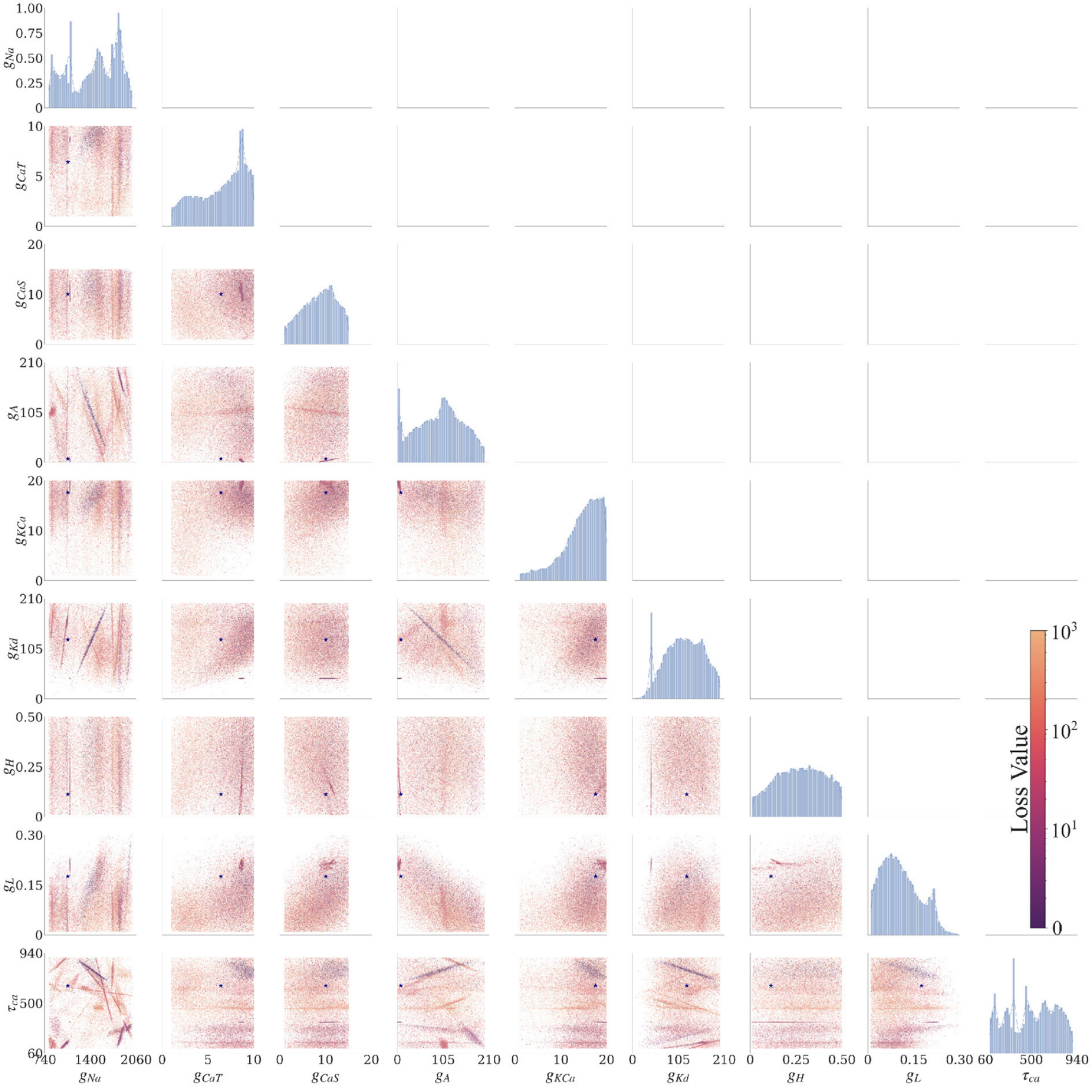
Final solution map of the posterior for the 9 parameters of the AM model for the individual constraint of the 0.0 nA injected current. The diagonal plots show the 1D marginals of each parameter, where the x-axis is the search range of the parameter, and the y-axis is the normalized distribution. The remaining plots are the 2D marginals of pairs of parameters, where x- and y-axes are the range of the pairs of parameters. Each point within each plot is a sample found by the MCMC algorithm. The colors indicate the associated loss value of this sample, where the purple color indicates a lower loss value and the yellow color indicates a higher loss value. Density of points will contribute to the darkness of an area. The more pronounced a color signifies that more points are overlaid on top of each other. For example, in the top left 2D marginal [*g*_*CaT*_, *g*_*Na*_] the dark purple area around [*g*_*CaT*_, *g*_*Na*_]~[9, 1200] is an accumulation of many overlaid points. This density can also be traced back to the 1D marginals to the top and the left side. The ground truth values are plotted as a purple asterisk for comparison to low loss modes of the parameter spaces.

The AM model has nine uncertain parameters that we want to infer; therefore, the posterior is a distribution in a nine-dimensional space. To visualize the nine-dimensional space, we consider one or two parameters at a time, where the remaining parameter dimensions are marginalized (i.e., summed up). The plots in Figure 3 show the solution maps that visualize the posterior. The denser regions within Figure 3 are parameter value sets that return lower loss function values; therefore they represent better fits between data and model outputs.

Along the diagonal of Figure 3 are the histograms for the (1D marginal) distribution for each parameter. For the 2D marginals in the lower triangle of Figure 3, the points have the opacity set to darker where their density is higher. This setup allows for easier visualization of trends within the posterior distribution with regard to parameter values. Note that the denser points for a particular parameter (*g*_*Na*_, for example) are not necessarily in the denser regions for another parameter (*g*_*Kd*_, for example), if one considers different plots. Such links between two parameters can be established only when *g*_*Na*_, *g*_*Kd*_ are plotted along the two axes of the same 2D marginal. In other words, a solution set containing parameter values selected from the densest distributions of each parameter will not necessarily yield a good solution.

#### 3.2.2. AM model—Individual constraint

We ran MCMC sampling on the AM model using individualized currents (0.0–0.4 nA at increments of 0.1 nA). This dataset is called the “Individual Constraint.” We also ran the MCMC search as a single aggregated search with all currents at once, which is discussed in a subsequent section. Below we present results from the individual constraints.

MCMC sampling produced solution maps of the posteriors for each of the individual currents (see Figure 3 for current 0.0 nA and the Supplementary Figures for all other currents). Within each of the maps one can search for the parameter set (i.e., a sample of MCMC) with the lowest loss value. Table 2 presents the results of such a search and one can consider the values listed in this table to be the optimal parameters, as recovered by MCMC. However, in addition to producing parameter sets with low loss values, the MCMC algorithm is able to recover the posterior distribution for all 9 parameters of the model within the search range for each injected current (0.0 to 0.4 nA, 0.1 nA increments).

**Table 2:** Parameter sets for the lowest loss values calculated by the MCMC algorithm.

**Injected** **current (nA)**	***g*_*Na*_**	***g*_*CaT*_**	***g*_*CaS*_**	***g*_*A*_**	***g*_*KCa*_**	***g*_*Kd*_**	***g*_*H*_**	***g*_*L*_**	**τ_*ca*_**	**Loss**
Uniform priors	[800, 2000]	[1, 10]	[1, 15]	[1, 200]	[1, 20]	[1, 200]	[0.01, 0.5]	[0.01, 0.5]	[100, 900]	–
0.0	1484.10	9.54	11.07	78.73	18.13	144.03	0.027	0.119	767.54	0.0008
0.1	1543.74	9.90	14.27	73.17	19.74	171.16	0.067	0.149	768.77	0.0002
0.2	1768.69	6.64	13.52	118.36	18.01	68.42	0.096	0.075	739.20	0.0348
0.3	1461.32	6.63	10.14	86.07	10.41	62.39	0.425	0.193	452.63	0.0013
0.4	1973.44	4.06	9.93	168.56	1.24	29.14	0.221	0.183	206.94	0.0047
Aggregate	1335.84	9.18	14.97	102.72	18.84	142.91	0.363	0.163	802.44	8.8109
Ground truth	1076.39	6.41	10.05	8.04	17.58	124.09	0.113	0.176	653.50	–

The range of the parameter priors is also provided in the first row. The units for the conductances are in *pS*/μ*m*^2^.

We summarized these findings in an arrangement of plots in Figure 3 for 0.0 nA injected current and for currents 0.1–0.4 nA in the Supplementary material. The rows and columns of subplots are each associated with one of the parameters (*g*_*Na*_, *g*_*CaT*_, *g*_*CaS*_, *g*_*A*_, *g*_*KCa*_, *g*_*Kd*_, *g*_*H*_, *g*_*L*_, and τ_*Ca*_).^1^ The interpretations of each of the plotted marginal types are explained in the following:

##### 3.2.2.1. 1D marginals

The diagonal portion of the subplot matrix shows the one-dimensional (1D) marginals (distribution histograms) of the solutions for each of the individual parameters. The x-axis represents the range of values, and the y-axis represents the probability of that value producing a good fit between data and model outputs. These distributions provide both the likeliest solutions (i.e., the peaks) as well as their uncertainty (distribution around the peaks). For example, the top left 1D marginal of the sodium conductance *g*_*Na*_ has 4 peaks, and the width of these peaks allows to assess the uncertainty (or sensitivity) of the sodium conductance *g*_*Na*_.

##### 3.2.2.2. 2D marginals

The remaining subplots are the two-dimensional (2D) marginals of the posterior distributions. Each row and column represent a parameter θ of the model, and each of the points within the subfigure is a solution found by the MCMC algorithm. To show the probability distribution within the solution space, we set the alpha value (the transparency of the color) of each solution to α = 10^−3^ (where opaque is α = 1). A highly probable region has many more points and therefore will appear more opaque. The color of each point represents the loss value ranging from blue to yellow (low to high, respectively). These plots show the dependence of each parameter on another. Some of these dependencies are linear (for example, [row, col]: [*g*_*Kd*_, *g*_*Na*_]), and some are non-linear (for example, [row, col]: [*g*_*Kd*_, *g*_*CaT*_]). Some of the linear dependencies are vertical and horizontal, signifying that one parameter is independent from the other (for example, vertical [*g*_*H*_, *g*_*CaT*_], [*g*_*H*_, *g*_*Na*_] and horizontal [τ_*Ca*_, *g*_*Cas*_]).

#### 3.2.3. AM model—Aggregate constraint

To test the versatility of the MCMC algorithm at solving the inverse problem for HH-type equations, we combined all the input currents into a single analysis called “aggregate constraint” (more details given in Section methods).

**Figure 4:**
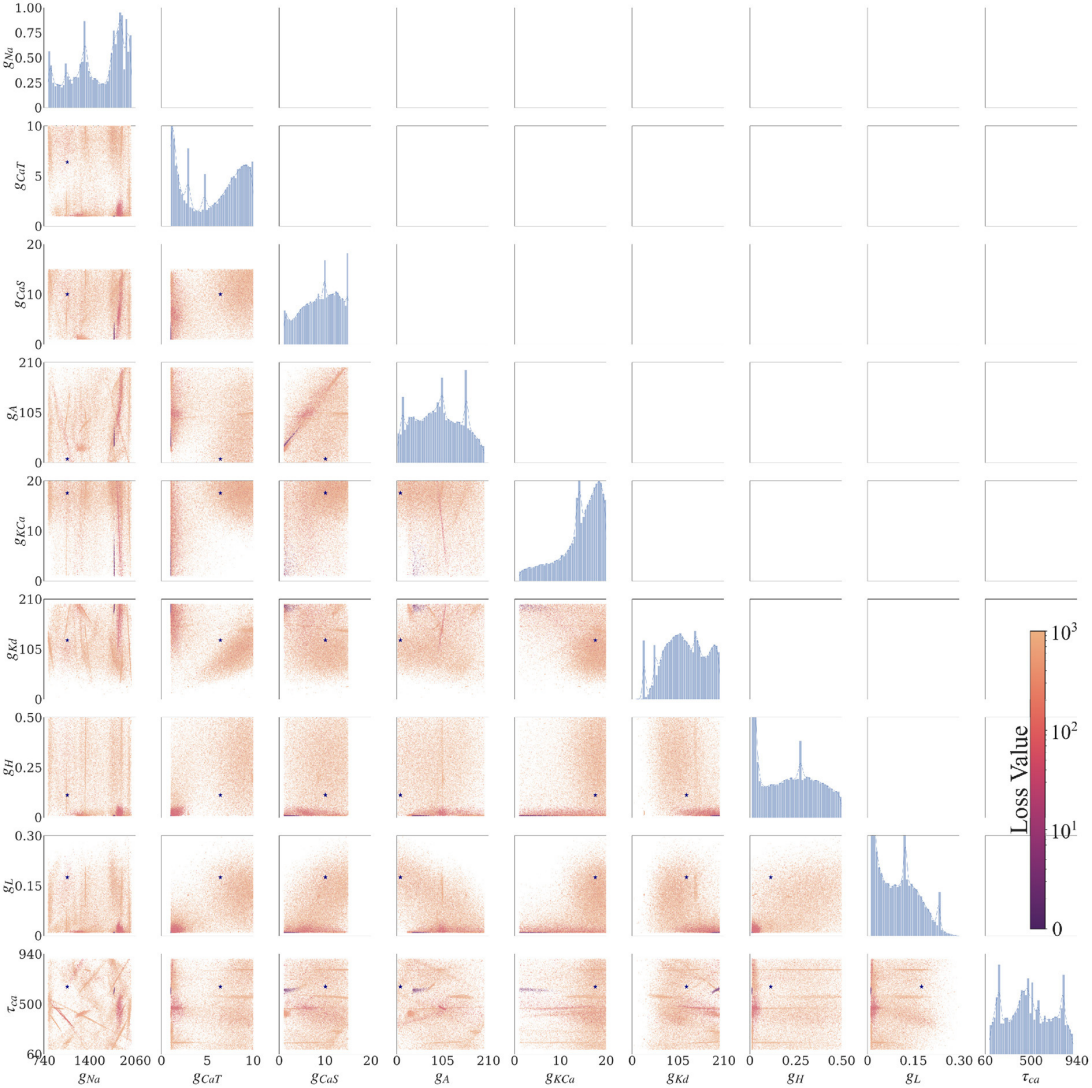
Solution maps of the posterior for the 9 parameters of the AM model for the aggregate constraint. The diagonal plots show the 1D marginals of each parameter. The x-axis is the search range of the parameter, and the y-axis is the normalized distribution. The remaining plots are the 2D marginals of pairs of parameters. The x- and y-axes are the range of the pairs of parameters. Each point within each plot is a sample found by the MCMC algorithm. The colors indicate the associated loss value of this sample, where purple color indicates a lower loss value and yellow color indicates a higher loss value. The density of the points will contribute to the darkness of an area. More pronounced color signifies that more points are overlaid on top of each other. This density can also be traced back to the 1D marginals to the top and the left side. The ground truth values are plotted as a purple asterisk for comparison to low loss modes of the parameter spaces.

**Figure 5:**
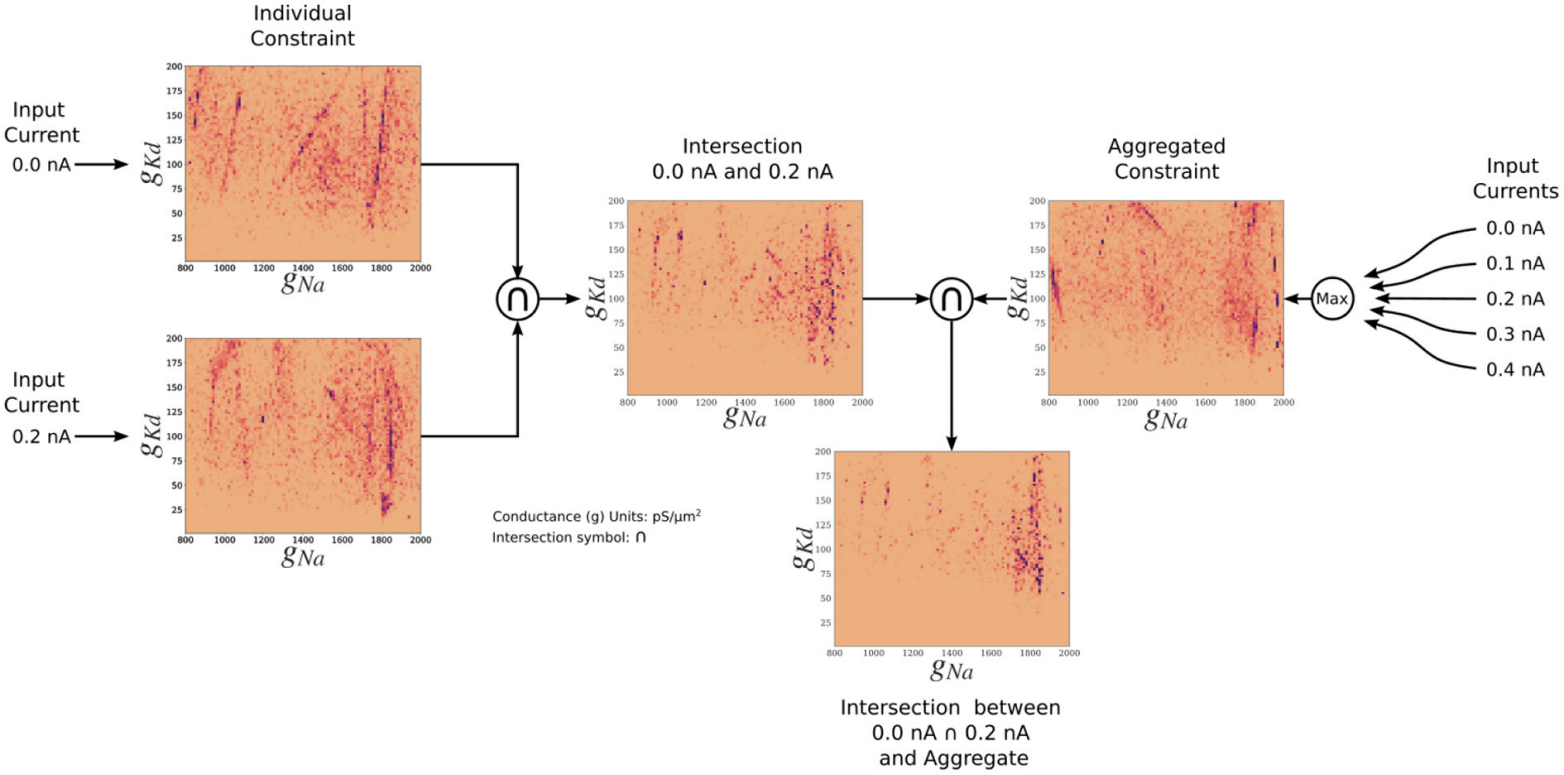
Two-dimensional (*g*_*Na*_ and *g*_*Kd*_) kernel density estimates (KDEs) of the solution maps of different cases, for visualization of the relationship between the aggregate constraint and the individual constraints. The arrows indicate the flow of the operation. The KDEs of the 0.0 and 0.2 nA individual constraints are on the left. The KDE of the aggregate constraint is shown on the right. The intersection is the elementwise multiplication of two or more KDEs. The final intersection between 0.0, 0.2 nA, and aggregate constraint shows the contribution of the 0.0 and 0.2 nA individual constraints to the aggregate constraint. The colormap scales have been adjusted so that values are visible.

The posterior resulting from the aggregate constraint analysis is visualized as a solution map in Figure 4. The same analysis as for the individual constrained can be performed to determine the peaks and variations of individual parameters using the 1D marginals and the dependence of two parameters using the 2D marginals. Compared with the individual constraint, the loss values are higher; hence the yellow colors dominate in these maps. The aggregate constraint solution map encompasses a subset of the individual constraint solution maps. To illustrate this in Figure 5, we show the intersection (multiplication of the posterior) of the kernel density estimates (KDEs) of the solution maps from the aggregate constraint and the two individual constraints 0.0 and 0.2 nA, as an example. Figure 5 first depicts the intersection between the individual constraints 0.0 and 0.2 nA, and compares it to the aggregate constraint *via* an additional intersection (elementwise multiplication) to extract their commonalities. The distinctive features (in dark blue/purple color) found in the individual constraints are found in the intersection (bottom) even though they were not prominent in the aggregate constraint results. In the next section we formalize the commonalities between solution maps, which we hinted at with intersections, using a metric called the Wasserstein distance.

#### 3.2.4. AM model—Distances between posterior distributions

To demonstrate the differences between the posterior distributions of the AM model between an individual constraint and the aggregate constraint, we computed the approximate Wasserstein distance for each individual injected current's posterior distribution against the aggregate constraint's posterior distribution. We carried this out for the posteriors with respect to all nine parameters as well as individual parameters (*via* marginals as depicted along the diagonals of Figures 3, 4). The approximate Wasserstein distance provides a quantitative metric of the total cost required to transform one probability distribution to another probability distribution (Givens and Shortt, 1984). The results are displayed in Table 3, with the rightmost column showing the Wasserstein distance of the entire posterior distribution (all nine parameters used) for a specific injected current. Overall, the posterior distribution of no injected current (0.0 nA) yields the closest (by Wasserstein distance) posterior distribution to the aggregate constraint, followed by 0.2 nA, then 0.1 and 0.3 nA.

**Table 3:** Normalized (using the chosen bounds of each parameter) Wasserstein distances for each posterior of individually injected currents against the aggregated constraint's posterior.

**Injected** **current (nA)**	**1D Wasserstein distance (WD)**									**9D WD**
	***g*_*Na*_**	***g*_*CaT*_**	***g*_*CaS*_**	***g*_*A*_**	***g*_*KCa*_**	***g*_*Kd*_**	***g*_*H*_**	***g*_*L*_**	**τ_*ca*_**	**All parameters**
0.0	0.0372	0.0909	0.0194	0.0231	0.0266	0.0306	0.1173	0.0359	0.0150	0.5264
0.1	0.0382	0.0762	0.0319	0.0788	0.0567	0.0437	0.1195	0.0396	0.0327	0.5319
0.2	0.0355	0.1139	0.0245	0.0926	0.0722	0.0302	0.1197	0.0525	0.0435	0.5690
0.3	0.0497	0.0582	0.0275	0.0975	0.0293	0.0267	0.0886	0.1076	0.0223	0.5335
0.4	0.0298	0.0816	0.0283	0.0934	0.0193	0.0594	0.0708	0.1074	0.0414	0.5375
aggregate	0.0	0.0	0.0	0.0	0.0	0.0	0.0	0.0	0.0	0.0

The distances are organized according to the dimensionality of the Wasserstein distance used: Wasserstein distances for single parameters posteriors use the one-dimensional (1D) distance while “all parameters” use the nine-dimensional (9D) distance. The 9D distance includes the entire nine-dimensional posterior as a group rather than the one-to-one comparisons of the 1D distance. The final row shows the Wasserstein distances for the aggregate current constraint. All these values are zero as it is the Wasserstein distance to oneself.

The parameter's posterior distributions that are the most different between an individual constraint and the aggregate constraint are the distributions for the parameter *g*_*H*_ and to a lesser extent for *g*_*CaT*_, *g*_*A*_, *g*_*L*_. This observation for the Wasserstein distances is consistent with the 1D marginal solution maps of the posterior when comparing the aggregate constraint in Figure 4 with Figure 3 and the additional solution maps of the posterior for other individual currents in the Supplementary material.

## 4. Discussion

Alonso and Marder (2019) presented a complex and realistic neurological model and proposed visualization techniques in order to help understand how different parameter sets can have similar model outputs. As did, Van Geit et al. (2007), Druckmann et al. (2007), and Prinz et al. (2003), they identified the challenge to find multiple parameter sets that are optimal in the sense that they generate good fits between data and model outputs. Consequently, this raised the need to develop algorithmic approaches in order to find these multiple sets.

In the present work, we design the inverse problem in a Bayesian framework, where multiple optimal parameter sets are part of a single multimodal posterior distribution. Furthermore, we utilize parallel tempering MCMC in order to recover the multiple modes in the posterior and visualize them in the solution maps. As a result, we have filled an important need in the field of neuroscience.

### 4.1. Interpreting solution maps and choosing parameter sets

The solution space that the MCMC algorithm provides (the solution maps) can be overwhelming especially when one is familiar with the classical gradient/optimization approach to an inverse problem (i.e., a single solution). Instead, our Bayesian framework provides continuous ranges of solutions from which one can select individual parameter value sets. We present two viewpoints as methods to interpret these results, the *single-solution viewpoint*, and the *multimodal viewpoint*.

#### 4.1.1. Single-solution viewpoint

As detailed in the Section results, one parameter set amongst all the posterior samples produces the lowest loss value (see Table 2). At the simplest interpretation, one could choose this parameter set as the solution to use. However, the Section results demonstrates that there are many parameter sets that provide low loss values which are close to the lowest value. For instance, we found, for the injected current of 0.0 nA, a total of 889 parameters are within 0.5% of the best parameter set and a total of 1,595 parameter samples are within 1% of the lowest loss (see Table 4). Of course, the precise numbers are dependent upon the number of MCMC iterations, but it shows that vast amounts of parameters are able to reproduce a given observational data as it is scored by the loss function. It is important to note that the loss function plays a crucial role here in measuring the fitness between data and model outputs (for more details, see Section impact of loss function on recovered parameters).

**Table 4:** Number of solutions given the injected current as a function of percentage difference from the best solution.

**Injected** **current (nA)**	**0.5*%***	**1.0*%***
0.0	889	1,595
0.1	916	1,618
0.2	21	118
0.3	120	233
0.4	149	265

**Figure 6:**
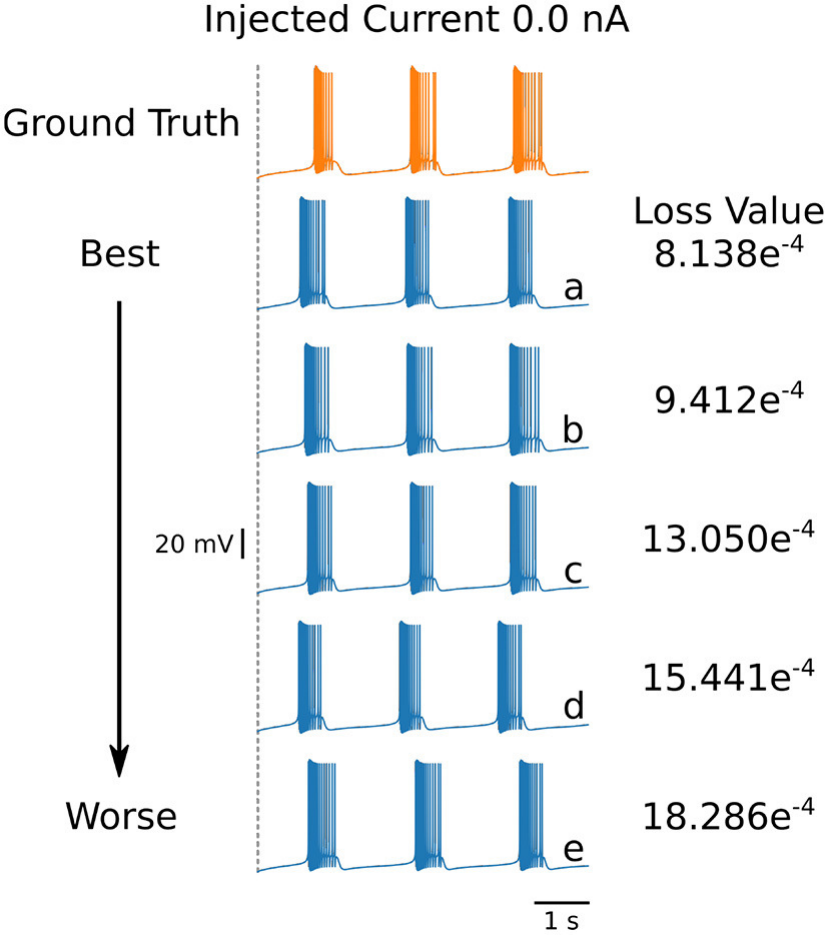
First 5 solutions for the injected current of 0 nA. These solutions are ranked from “best” to “worse” **(A–E)** according to their loss. Even a nearly-identical loss can produce a qualitatively different voltage trace, as shown by traces **(D,E)**. The values for the parameters sets for each of the voltage traces can be found in Table 5.

For instance, for a 0.0 nA injected current, 889 solutions are 0.5% away from the best 0.0 nA solution. MCMC is able to find these multiple acceptable solutions. Figure 6 visualizes an example set of voltage traces generated from these solution parameter sets.

**Table 5:** Parameters for the first 5 solutions for the injected current 0.0 nA. The voltage traces for each of these parameters sets are shown in Figure 6.

**Figure 6** **trace**	**Injected** **current (nA)**	***g*_*Na*_**	***g*_*CaT*_**	***g*_*CaS*_**	***g*_*A*_**	***g*_*KCa*_**	***g*_*Kd*_**	***g*_*H*_**	***g*_*L*_**	**τ_*ca*_**	**Loss**
a	0.0	1484.995	9.543	11.072	78.728	18.135	144.027	0.027	0.119	767.541	8.138e^−4^
b	0.0	1554.694	8.060	13.169	60.339	19.039	165.425	0.192	0.187	728.020	9.412e^−4^
c	0.0	1578.022	9.642	11.247	43.430	17.730	175.763	0.341	0.214	726.248	13.050e^−4^
d	0.0	1587.899	9.786	11.207	39.391	18.545	178.563	0.104	0.180	723.591	15.441e^−4^
e	0.0	1526.351	9.988	11.957	63.573	16.147	158.435	0.446	0.214	747.016	18.286e^−4^

One can also locate these solutions in some of purple bands found in Figure 3 (i.e., [*g*_*Na*_, τ_*Ca*_] = [1485, 767] for Trace a).

Even though the loss values are small for the parameters in Table 4, the voltage traces produced by these sets are slightly different. To illustrate these differences, we plot in Figure 6 the first five voltage traces in the order of the loss value from best to (slightly) worse with respect to the ground truth shown (shown in orange color). The change in loss value is smaller than 10^−2^ and nearly identical for the last two traces (d) and (e). However, these traces visually appear to be somewhat different, thus illustrating that different parameter sets may yield similar loss values while producing potentially different traces when inspected by eye. It highlights the crucial role of the loss function, and it shows that it is important for physiologist to inspect the solution maps, visualizing the posterior, to further investigate or constrain parameter ranges.

#### 4.1.2. Multimodal viewpoint

Because of the preceding observations, it is beneficial to look for multiple modes or parameter value regions (rather than a single point) in the posterior: this is illustrated in the 1D and 2D marginals described in the Section results. In the 1D marginals, one can assess important parameter values within a range by the different peaks observed within the distribution as well as its sensitivity by the width/spread under peaks. Furthermore, the limitation of parameter bounds defined by the prior can be determined, if distributions appear to abruptly stop at the boundaries. For instance, in the 1D marginal for parameter *g*_*KCa*_ found in Figure 3, it appears that the limits of the range for *g*_*KCa*_ values may have been too small as the distribution begins a descent at the upper bound. Such insight can lead the modeler to reconsider or investigate the range of the parameters. The 2D marginals help to determine which parameters are correlated (i.e., trade-off between each other). Taken together, one can begin to look at solution areas that previously were left unexplored, perhaps opening new avenues for investigation. This possibility allows for investigators to be able to examine a selection of parameter value set solutions found, rather than just utilizing the parameter value set of the lowest loss solution.

We now recall the ground truth values of the parameters that we used in the numerical experiments (see Section methods) and compare them to the plots of the posteriors in Figures 3, 4. We note that the parameter values used to generate the ground truth do not always correspond to the areas under the peaks in the marginals of the plotted solution maps of the posterior. For instance, the parameter values of *g*_*Na*_, *g*_*H*_, and *g*_*L*_ used for the ground truth do not correspond to areas of high density in the solution maps. We believe one possible reason for this is that some of the ground truth parameter values give rise to models that are less robust compared with the parameter values where peaks in the posterior are higher and/or wider as recovered by MCMC. Since the presented algorithm provides parameter sensitivities and the solution maps of the posterior enable to interpret these sensitivities, an evaluation of the robustness of estimated parameters is possible. This can lead to potentially important insights toward understanding neuron models.

### 4.2. Impact of loss function on recovered parameters

**Figure 7:**
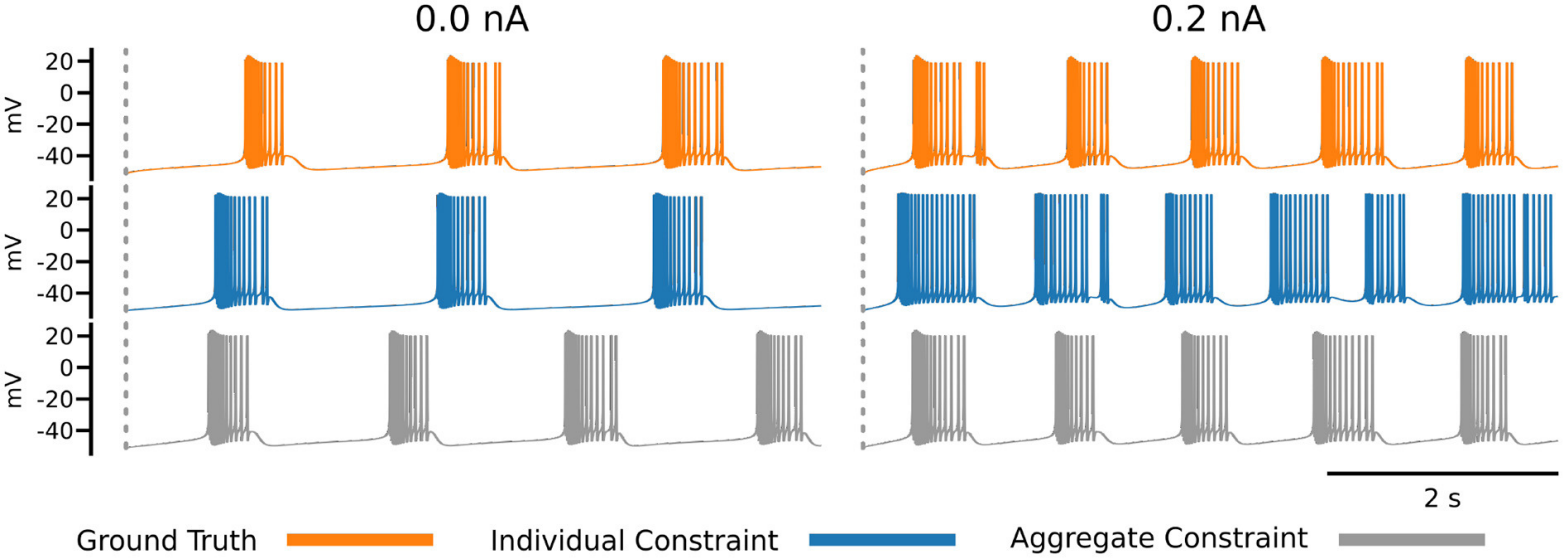
Each row of this figure shows the voltage traces for the ground truth (top orange), for the best solution sets given the individual constraints (middle blue) and the best solution set given the aggregate constraint (bottom gray). Each column shows the traces for different injected currents. Loss values for these parameter solution sets are 0.0008 for the 0.0 nA individual constraint, 0.0348 for the 0.2 nA individual constraint, and 8.8109 for the aggregate constraint.

The impact of the choice of loss function becomes apparent in Figure 7, which shows the ground truth voltage trace generated from the AM model with the ground truth set of parameters (orange color). This trace serves as the data in our inverse problem. To measure the fit between data and model outputs, we consider a particular loss function that is derived from the loss function employed by Alonso and Marder (2019). Comparing the orange (ground truth) and the blue trace graphs, we observe discrepancies between the traces that may appear as inadequate fits from a physiological perspective. These discrepancies are caused by the loss function, which defines a distance metric between traces. In essence, a possible limitation of the chosen loss function is that it quantifies disparate voltage traces as too similar by giving it a low loss value. This demonstrates the difficulty of finding appropriate losses for inverse modeling in neuroscience and is one main reason for manual and heuristic approaches for solving inverse problems governed by neural dynamics models.

To address this issue, Alonso and Marder (2019) invested efforts to refine the loss to specific inference setups that are targeted. This approach can be prone to human bias and is labor intensive. An alternative approach is taken in the present work, because we aim to automate the inference as much as possible. The idea is to augment the measure for fitting data and model outputs with additional information. In this work, we proposed to augment the loss function with additional voltage traces that are generated at different input currents. This approach results in the inverse problem setup with aggregated current constraint, and we observe traces from solution sets using this constraint are better than the individual constraint for some currents (gray trace at 0.2 nA). An alternative approach to the aggregate current constraint would be to design new loss functions (for individual currents), which is direction for future work.

### 4.3. Physiological meaning and future opportunities

Extracting physiological information from the solution maps is the ultimate goal of this exercise. For instance, one can rank the importance of a parameter by looking at the 2D marginals. Looking at the bottom row of Figure 3, we observe that τ_*Ca*_ is highly correlated with *g*_*Kd*_ while *g*_*CaT*_ and *g*_*CaS*_ are more independent of τ_*Ca*_ for the injection current constraint 0.0 nA. Therefore, the analysis shows that the calcium component of the afterhyperpolarization (AHP) needed to replicate the ground truth spiking traces is dominated by the time constant τ_*Ca*_.^2^

Alonso and Marder (2019) showed that multiple parameter estimations exist for solving one neuronal dynamics model. Our framework complements this finding by providing one possible method for which a larger sets of parameters can be found that reproduce desired model behavior. We believe that the observed multimodal posterior is the norm rather than the exception in the field of neuroscience. As models increase in complexity (and therefore increase in parameters), the likelihood of a multimodal solution will increase. For instance, although studies of variability of neuronal behavior have concentrated on the role of ion channel density (e.g., Marder and Goaillard, 2006), changes in the voltage dependence of channels resulting from changes in phosphorylation (Park et al., 2006) or the binding of accessory proteins (Bosch et al., 2015) are likely to be equally important. The advantage of Bayesian inference together with MCMC is that these additional variables can be taken into account.

The current MCMC sampling setup operates within the bounds based on our prior assumptions from general knowledge, literature review, or colleagues. Another viewpoint of our results is that our MCMC-based Bayesian framework provides the ability to “test” these prior assumptions using the experimental data and system model by looking at the shape of the posterior, as mentioned above. Peaks at the boundaries of the ranges are likely to indicate too narrow a range of sampled parameter values (see parameters *g*_*A*_ and *g*_*KCa*_ in Figure 3). This could lead experimentalists to look beyond their prior assumptions.

Finally, this method has the potential to help beyond ion channel parameter inference as was done here with the AM model. As observed in the literature, it remains a fundamental challenge to find viable sets of parameters for compartmental models, for instance, see Hay et al. (2013) for pyramid cell models and Zang et al. (2018) for Purkinje cell models. An even greater challenge is the description of the parameters spaces, which was noted to be important (Hay et al., 2013). The present work shows a potential pathway to addressing these challenges for such complex compartmental models. It should be noted as well that simulators, such as NEURON (Carnevale and Hines, 2006), can be used to generate the traces to feed into the loss function and parallel tempering MCMC. Overcoming these challenges in the future would bridge an important gap that is currently present in neuroscience: the gap between the inference with model-generated ground truth data and the inference with experimentally observed (Van Geit et al., 2007) data of complex (compartmental) models.

## Data availability statement

The raw data supporting the conclusions of this article will be made available by the authors, without undue reservation. The output codes and models of this work are available in ModelDB with accession number 267583.

## Author contributions

MC, YW, and JR contributed to the conception and design of the study. JV, GI, JR, YW, and MC wrote the source code and conducted the numerical experiments. CH, RP, and NS assisted with interpreting the results and statistical analysis. All authors contributed to sections of the manuscript. All authors contributed to the article and approved the submitted version.

## Funding

The efforts of MC, CH, RP, and NS were supported in part by the National Institutes of Health under Award No. R01NS109552 and NINDS R01NS125863. The effort of GI was supported by the NSF Mathematical Sciences Graduate Internship. The effort of JR was supported in part by the U.S. Department of Energy, Office of Science, under contract DE-AC02-06CH11357. The effort of YW was supported in part by the Center for Energy and Sustainability at California State University, Los Angeles, and the National Science Foundation under Award No. HRD-1547723.

## Conflict of interest

The authors declare that the research was conducted in the absence of any commercial or financial relationships that could be construed as a potential conflict of interest.

## Publisher's note

All claims expressed in this article are solely those of the authors and do not necessarily represent those of their affiliated organizations, or those of the publisher, the editors and the reviewers. Any product that may be evaluated in this article, or claim that may be made by its manufacturer, is not guaranteed or endorsed by the publisher.

## Footnotes

^1^Figure 3 resembles a triangular matrix, where the upper right triangle is omitted because it is symmetric to the lower left triangle.

^2^Note that to be able to reach such a conclusion with (more traditional) optimization techniques that deliver point estimates, a sensitivity analysis would be required in addition to finding an optimum.

## References

[B1] Achard P. De Schutter E. (2006). Complex parameter landscape for a complex neuron model. PLoS Comput. Biol. 2, e94. 10.1371/journal.pcbi.0020094 16848639 PMC1513272

[B2] Ahmadian Y. Pillow J. W. Paninski L. (2011). Efficient Markov chain Monte Carlo methods for decoding neural spike trains. Neural Comput. 23, 46–96. 10.1162/NECO_a_00059 20964539 PMC4740351

[B3] Alonso L. M. Marder E. (2019). Visualization of currents in neural models with similar behavior and different conductance densities. eLife 8, e42722. 10.7554/eLife.42722 30702427 PMC6395073

[B4] Amarasingham A. Geman S. Harrison M. T. (2015). Ambiguity and nonidentifiability in the statistical analysis of neural codes. Proc. Natl. Acad. Sci. U.S.A. 112, 6455–6460. 10.1073/pnas.1506400112 25934918 PMC4443375

[B5] Ballnus B. Hug S. Hatz K. Garlitz L. Hasenauer J. Theis F. J. (2017). Comprehensive benchmarking of Markov chain Monte Carlo methods for dynamical systems. BMC Syst. Biol. 11, 63. 10.1186/s12918-017-0433-1 28646868 PMC5482939

[B6] Bittner S. R. Palmigiano A. Piet A. T. Duan C. A. Brody C. D. Miller K. D.. (2021). Interrogating theoretical models of neural computation with emergent property inference. Elife. 10, e56265. 10.1101/837567 34323690 PMC8321557

[B7] Bosch M. K. Carrasquillo Y. Ransdell J. L. Kanakamedala A. Ornitz D. M. Nerbonne J. M. (2015). Intracellular FGF14 (iFGF14) is required for spontaneous and evoked firing in cerebellar Purkinje neurons and for motor coordination and balance. J. Neurosci. 35, 6752–6769. 10.1523/JNEUROSCI.2663-14.2015 25926453 PMC4412895

[B8] Buhry L. Grassia F. Giremus A. Grivel E. Renaud S. Sanghi S. (2011). Automated parameter estimation of the Hodgkin-Huxley model using the differential evolution algorithm: application to neuromimetic analog integrated circuits. Neural Comput. 23, 2599–2625. 10.1162/NECO_a_00170 21671785

[B9] Caranica C. Al-Omari A. Deng Z. Griffith J. Nilsen R. Mao L.. (2018). Ensemble methods for stochastic networks with special reference to the biological clock of *Neurospora crassa*. PLoS ONE 13, e0196435. 10.1371/journal.pone.0196435 29768444 PMC5955539

[B10] Carnevale N. T. Hines M. L. (2006). The NEURON Book. Cambridge, UK: Cambridge University Press. 10.1017/CBO9780511541612

[B11] Chen Z. (2013). An overview of Bayesian methods for neural spike train analysis. Comput. Intell. Neurosci. 2013, 1–17. 10.1155/2013/251905 24348527 PMC3855941

[B12] Conda-Forge Community. (2015). The conda-forge Project: Community-based Software Distribution Built on the conda Package Format and Ecosystem. Zenodo. 10.5281/zenodo.4774216

[B13] Doi S. Onoda Y. Kumagai S. (2002). Parameter estimation of various Hodgkin–Huxley-type neuronal models using a gradient-descent learning method, in Proceedings of the 41st SICE Annual Conference (IEEE) (Osaka), 1685–1688.

[B14] Doruk R. O. Abosharb L. (2019). Estimating the parameters of FitzHugh–Nagumo neurons from neural spiking data. Brain Sci. 9, 364. 10.3390/brainsci9120364 31835351 PMC6956007

[B15] Druckmann S. Banitt Y. Gidon A. A. Schürmann F. Markram H. Segev I. (2007). A novel multiple objective optimization framework for constraining conductance-based neuron models by experimental data. Front. Neurosci. 1, 7–18. 10.3389/neuro.01.1.1.001.2007 18982116 PMC2570085

[B16] Geyer C. J. Thompson E. A. (1995). Annealing Markov chain Monte Carlo with applications to ancestral inference. J. Am. Stat. Assoc. 90, 909–920. 10.1080/01621459.1995.10476590

[B17] Givens C. R. Shortt R. M. (1984). A class of Wasserstein metrics for probability distributions. Michigan Math. J. 31, 231–240. 10.1307/mmj/1029003026

[B18] Golowasch J. Goldman M. S. Abbott L. F. Marder E. (2002). Failure of averaging in the construction of a conductance-based neuron model. J. Neurophysiol. 87, 1129–1131. 10.1152/jn.00412.2001 11826077

[B19] Gonçalves P. J. Lueckmann J.-M. Deistler M. Nonnenmacher M. Ocal K. Bassetto G.. (2020). Training deep neural density estimators to identify mechanistic models of neural dynamics. eLife 9, e56261. 10.7554/eLife.56261 32940606 PMC7581433

[B20] Gupta S. Lee R. E. C. Faeder J. R. (2020). Parallel tempering with Lasso for model reduction in systems biology. PLoS Comput. Biol. 16, e1007669. 10.1371/journal.pcbi.1007669 32150537 PMC7082068

[B21] Hartoyo A. Cadusch P. J. Liley D. T. J. Hicks D. G. (2019). Parameter estimation and identifiability in a neural population model for electro-cortical activity. PLoS Comput. Biol. 15, e1006694. 10.1371/journal.pcbi.1006694 31145724 PMC6542506

[B22] Hastings W. K. (1970). Monte Carlo sampling methods using Markov chains and their applications. Biometrika 57, 97–109. 10.1093/biomet/57.1.97

[B23] Hay E. Schürmann F. Markram H. Segev I. (2013). Preserving axosomatic spiking features despite diverse dendritic morphology. J. Neurophysiol. 109, 2972–2981. 10.1152/jn.00048.2013 23536715

[B24] Heckman C. J. Mottram C. Quinlan K. Theiss R. Schuster J. (2009). Motoneuron excitability: the importance of neuromodulatory inputs. Clin. Neurophysiol. 120, 2040–2054. 10.1016/j.clinph.2009.08.009 19783207 PMC7312725

[B25] Hodgkin A. L. Huxley A. F. (1952). A quantitative description of membrane current and its application to conduction and excitation in nerve. J. Physiol. 117, 500–544. 10.1113/jphysiol.1952.sp004764 12991237 PMC1392413

[B26] Hultborn H. Pierrot-Deseilligny E. (1979). Input-output relations in the pathway of recurrent inhibition to motoneurones in the cat. J. Physiol. 297, 267–287. 10.1113/jphysiol.1979.sp013039 231651 PMC1458719

[B27] Łacki M. K. Miasojedow B. (2016). State-dependent swap strategies and automatic reduction of number of temperatures in adaptive parallel tempering algorithm. Stat. Comput. 26, 951–964. 10.1007/s11222-015-9579-0

[B28] Liu Z. Golowasch J. Marder E. Abbott L. (1998). A model neuron with activity-dependent conductances regulated by multiple calcium sensors. J. Neurosci. 18, 2309–2320. 10.1523/JNEUROSCI.18-07-02309.1998 9502792 PMC6793093

[B29] Mainen Z. F. Joerges J. Huguenard J. R. Sejnowski T. J. (1995). A model of spike initiation in neocortical pyramidal neurons. Neuron 15, 1427–1439. 10.1016/0896-6273(95)90020-9 8845165

[B30] Marder E. Goaillard J.-M. (2006). Variability, compensation and homeostasis in neuron and network function. Nat. Rev. Neurosci. 7, 563–574. 10.1038/nrn1949 16791145

[B31] Marinari E. Parisi G. (1992). Simulated tempering: a new Monte Carlo scheme. Europhys. Lett. 19, 451–458. 10.1209/0295-5075/19/6/002

[B32] Meliza C. D. Kostuk M. Huang H. Nogaret A. Margoliash D. Abarbanel H. D. I. (2014). Estimating parameters and predicting membrane voltages with conductance-based neuron models. Biol. Cybernet. 108, 495–516. 10.1007/s00422-014-0615-5 24962080

[B33] Metropolis N. Rosenbluth A. W. Rosenbluth M. N. Teller A. H. Teller E. (1953). Equation of state calculations by fast computing machines. J. Chem. Phys. 21, 1087–1092. 10.2172/4390578 41342507

[B34] Miasojedow B. Moulines E. Vihola M. (2013). An adaptive parallel tempering algorithm. J. Comput. Graph. Stat. 22, 649–664. 10.1080/10618600.2013.778779

[B35] Nadim F. Olsen O. De Schutter E. Calabrese R. (1995). Modeling the leech heartbeat elemental oscillator I. Interactions of intrinsic and synaptic currents. J. Comput. Neurosci. 2, 215–235. 10.1007/BF00961435 8521288

[B36] Nocedal J. Wright S. J. (2006). Numerical Optimization, 2nd Edn. New York, NY: Springer.

[B37] Pape H.-C. McCormick D. A. (1989). Noradrenaline and serotonin selectively modulate thalamic burst firing by enhancing a hyperpolarization-activated cation current. Nature 340, 715–718. 10.1038/340715a0 2475782

[B38] Park K.-S. Mohapatra D. P. Misonou H. Trimmer J. S. (2006). Graded regulation of the Kv2. 1 potassium channel by variable phosphorylation. Science 313, 976–979. 10.1126/science.1124254 16917065

[B39] Petzold L. (1983). Automatic selection of methods for solving stiff and nonstiff systems of ordinary differential equations. SIAM J. Sci. Stat. Comput. 4, 136–148. 10.1137/0904010

[B40] Prinz A. A. Billimoria C. P. Marder E. (2003). Alternative to hand-tuning conductance-based models: construction and analysis of databases of model neurons. J. Neurophysiol. 90, 3998–4015. 10.1152/jn.00641.2003 12944532

[B41] Prinz A. A. Bucher D. Marder E. (2004). Similar network activity from disparate circuit parameters. Nat. Neurosci. 7, 1345–1352. 10.1038/nn1352 15558066

[B42] René A Longtin A. Macke J. H. (2020). Inference of a mesoscopic population model from population spike trains. Neural Comput. 32, 1448–1498. 10.1162/neco_a_01292 32521212

[B43] Roffman R. C. Norris B. J. Calabrese R. L. (2012). Animal-to-animal variability of connection strength in the leech heartbeat central pattern generator. J. Neurophysiol. 107, 1681–1693. 10.1152/jn.00903.2011 22190622 PMC3311676

[B44] Rudi J. Bessac J. Lenzi A. (2021). Parameter estimation with dense and convolutional neural networks applied to the FitzHugh-Nagumo ODE, in Proceedings of Mathematical and Scientific Machine Learning (MSML21) (Lausanne), 1–27.

[B45] Schmutz V. Gerstner W. Schwalger T. (2020). Mesoscopic population equations for spiking neural networks with synaptic short-term plasticity. J. Math. Neurosci. 10, 5. 10.1186/s13408-020-00082-z 32253526 PMC7136387

[B46] Schulz D. J. Goaillard J.-M. Marder E. (2006). Variable channel expression in identified single and electrically coupled neurons in different animals. Nat. Neurosci. 9, 356–362. 10.1038/nn1639 16444270

[B47] Schulz D. J. Goaillard J.-M. Marder E. E. (2007). Quantitative expression profiling of identified neurons reveals cell-specific constraints on highly variable levels of gene expression. Proc. Natl. Acad. Sci. U.S.A. 104, 13187. 10.1073/pnas.0705827104 17652510 PMC1933263

[B48] Smith R. C. (2013). Uncertainty Quantification: Theory, Implementation, and Applications. Philadelphia, PA: Society for Industrial and Applied Mathematics.

[B49] Soto-Trevino C. Rabbah P. Marder E. Nadim F. (2005). Computational model of electrically coupled, intrinsically distinct pacemaker neurons. J. Neurophysiol. 94, 590–604. 10.1152/jn.00013.2005 15728775 PMC1941697

[B50] Städter P. Schälte Y. Schmiester L. Hasenauer J. Stapor P. L. (2021). Benchmarking of numerical integration methods for ODE models of biological systems. Sci. Rep. 11, 2696. 10.1038/s41598-021-82196-2 33514831 PMC7846608

[B51] Stapor P. Weindl D. Ballnus B. Hug S. Loos C. Fiedler A.. (2018). PESTO: Parameter EStimation TOolbox. Bioinformatics 34, 705–707. 10.1093/bioinformatics/btx676 29069312 PMC5860618

[B52] Swensen A. M. Bean B. P. (2005). Robustness of burst firing in dissociated purkinje neurons with acute or long-term reductions in sodium conductance. J. Neurosci. 25, 3509. 10.1523/JNEUROSCI.3929-04.2005 15814781 PMC6725377

[B53] Toth B. A. Kostuk M. Meliza C. D. Margoliash D. Abarbanel H. D. I. (2011). Dynamical estimation of neuron and network properties I: variational methods. Biol. Cybernet. 105, 217–237. 10.1007/s00422-011-0459-1 21986979 PMC5759962

[B54] Valderrama-Bahamo G. I. (2019). MCMC techniques for parameter estimation of ODE based models in systems biology. Front. Appl. Math. Stat. 5, 55. 10.3389/fams.2019.0005525066046

[B55] Van Geit W. Achard P. De Schutter E. (2007). Neurofitter: a parameter tuning package for a wide range of electrophysiological neuron models. Front. Neuroinform. 1, 1. 10.3389/neuro.11.001.2007 18974796 PMC2525995

[B56] Van Geit W. De Schutter E. Achard P. (2008). Automated neuron model optimization techniques: a review. Biol. Cybernet. 99, 241–251. 10.1007/s00422-008-0257-6 19011918

[B57] Vavoulis D. V. Straub V. A. Aston J. A. D. Feng J. (2012). A self-organizing state-space-model approach for parameter estimation in Hodgkin-Huxley-type models of single neurons. PLoS Comput. Biol. 8, e1002401. 10.1371/journal.pcbi.1002401 22396632 PMC3291554

[B58] Virtanen P. Gommers R. Oliphant T. E. Haberland M. Reddy T. Cournapeau D.. (2020). SciPy 1.0: fundamental algorithms for scientific computing in Python. Nat. Methods 17, 261–272. 10.1038/s41592-020-0772-5 32015543 PMC7056644

[B59] Vousden W. D. Farr W. M. Mandel I. (2016). Dynamic temperature selection for parallel tempering in Markov chain Monte Carlo simulations. Monthly Notices R. Astron. Soc. 455, 1919–1937. 10.1093/mnras/stv2422

[B60] Zang Y. Dieudonné S. De Schutter E. (2018). Voltage-and branch-specific climbing fiber responses in purkinje cells. Cell Rep. 24, 1536–1549. 10.1016/j.celrep.2018.07.011 30089264

