# Supplementary material for: Multimodal parameter spaces of a complex multi-channel neuron model
Source: Front Syst Neurosci. 2022 Oct 20;16:999531. doi: 10.3389/fnsys.2022.999531 (PMC9632740; doi:10.3389/fnsys.2022.999531)
Supplement: Supplementary file 3 [file Image_3.pdf]

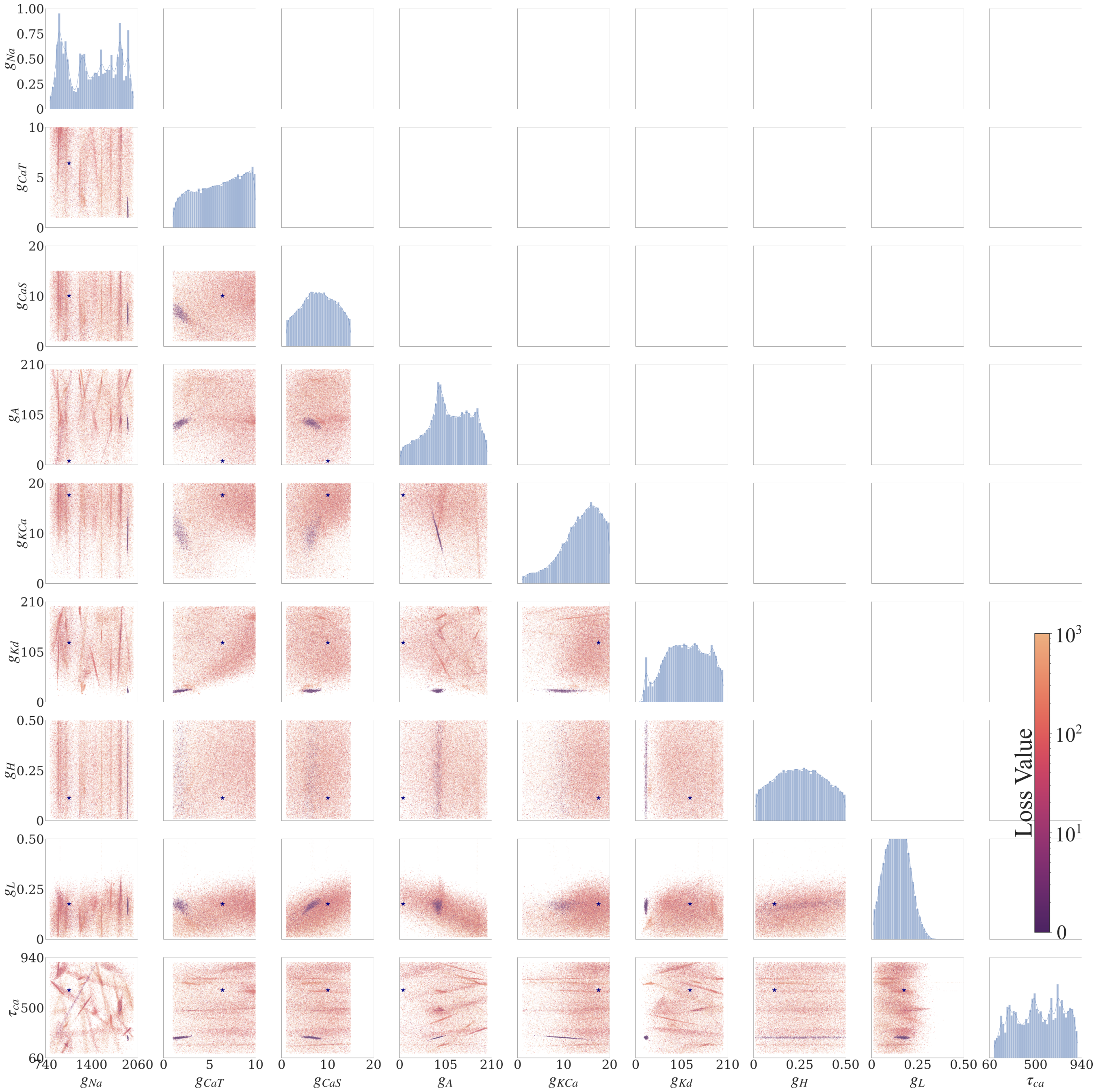


**Figure S3.** *0.3 nA results*: Diagonal shows the 1D marginals for each of the parameters. The remaining plots show the 2D marginal relating one parameter with an other. The color represents the loss value from blue to yellow (low to high). The density is represented by the opacity (i.e., light to dark shows low to high densities). Each point is set to a transparency level of $\alpha = 1e - 3$ where $\alpha = 1$ is opaque. As such, darker regions have more dots and indicated higher probabilities for good fits between data and model outputs.
